# Notes and News

**Published:** 1890-12-20

**Authors:** 


### Notes and News.

Collected and Communicated.

The Christmas card which the philanthropist should send to his hard-hearted neighbours is the Hon. Roden Noel's "Poor People's Christmas," a dainty little pamphlet in white and gold, containing in poetic form a story calculated to touch the most selfish. The pamphlet is only the price of an ordinary card of good wishes, but its worth is not demonstratable in pence, for it is sure to produce both thought and deed.

A substantial pamphlet reaches us from Melbourne—"the seventeenth annual report of the Hospital Saturday and Sunday Fund." The committee last year distributed £10,900; the expenses were £290. Of the receipts for the year, £3,521 2s. 11d. was contributed by employés in places of business, factories, government departments, and on public works, while £7,937 19s. 2d. was derived from collections in places of worship, Sunday and State schools, and various other sources.

"Truth" has done much to hold up the mirror to the faults and foibles of the world, more especially the philanthropical world. In its Christmas number there is some hard hitting for the devotees of hypnotism, under the title of "The Song of the Isms"—

Showing how each "Ism,"
Even as of yore,
Always is succeeded
By one Ism more.

Lady Stanley of Alderley last week opened what is somewhat absurdly termed a "Medical Institute" to the Women's Hospital in the Euston Road. The "Institute" consists of a pleasant study for the use of the lady medical students. The *Pall Mall Gazette* tells the following tale to the credit of this hospital: "It was about one of those ovariotomy cases which are perhaps the commonest occasion of overworking a nurse, as it is usual to keep the same one in attendance until a critical period—sometimes days and nights in length—is passed. The lady doctor, as doctors use, remarked to the matron, 'I shall want the same nurse to-night and to-morrow.' 'Oh, no,' was the reply; 'I couldn't think of letting my nurses be worked like that.' 'But they have always done it for me at ——.' 'I'm sorry—it's altogether wrong,' was the final reply."

The perpetual fogs of last week caused a rise in the deaths from lung diseases; for four days and nights the gas was not out in many of the London hospitals. But the typhoid epidemic is decreasing, and so is diphtheria. There is a story told of an American who arrived in London one evening last week; in the morning, the waiter called him, and drew up the blind; a faint yellow gloom showed through the window. "What is that?" demanded the American. "That, sir, is daylight," replied the waiter. "Then pull down the blind, and never let me see it again," said the American.

Preparations for Christmas are going on in both home and hospital, and those who wish the home festival to be happy will first give some thought to the hospital. A cheque, a parcel of clothing, a hamper of fruit, a bundle of holly: we can all do something for the sick at this time. And every town ought to look after its own hospitals and institutions; the ladies of Chichester, the gentlemen of Bristol, the children of Liverpool, are they all doing what they can for the hospitals in their towns? There ought not to be a single sick person on Christmas Day left without some little attention to show the universal good-will.

A strange community is that of Agua di Dios, the leper village in Colombia, as described by the British Consul at Bogota. Situated at about fourteen hundred feet above the sea level, with a dry sandy soil, and a temperature of 82 to 85 deg. Fahrenheit, this spot has been chosen for the lazaretto by the Government on account of its ancient reputation for the cure of leprosy. Some 520 sufferers from this terrible visitation dwell here, and form about one-third of the population; but the most remarkable fact regarding the settlement is that lepers and healthy persons are described as living on terms of perfect intimacy, there being no specific leper quarter, though every house in the village stands apart in a garden. Our Consul states that there is no case on record of the disease having been contracted here by contagion. Even where lepers have married healthy persons, the husband or wife has never been known to take it from the other. On the other hand, the fact is admitted that children born of such unions are generally afflicted.

The Hospital Reform Inquiry at Birmingham has come to an end; the last day was occupied by discussing an interesting provident dispensary scheme. The Rev. Joseph Wood, who was formerly a member of the committee of the Leicester Provident Dispensary and Infirmary Board, stated that out of a population of 150,000 at Leicester the dispensary possessed 35,000 members. There was one hospital in the town fairly well supported on an income of £12,000 a-year, while it had 12,000 out-patients. This showed one in twelve of the population compared with one in three in Birmingham. They attributed the difference entirely to the presence of a large provident dispensary. The head of a family paid a subscription of one penny per week, and an additional penny was charged for the wife and all children under fourteen years of age. From this source an income of £5,500 was obtained, while the total income amounted to £6,642. About £400 came from subscriptions. Out of the income there was provided and paid for the working expenses of a central infirmary and seven branch infirmaries. There were twenty medical men appointed to the various dispensaries, and the members had the privilege of choosing any medical man they might like. The payment to the medical staff averaged £150 each per annum—the payment varying from £30 and £40 to £500 each. It seemed to him that such a plan as this could be applied to Birmingham if they had the courage to deal with the matter. Mr. Hodges, the secretary of the General Provident and Benevolent Institution, gave some interesting information. He explained that while the bulk of the members were *bona fide* work-people, there was a good sprinkling of "decent people."

The following vacancies are announced this week, the amount of the salary in each case being given after the name of the institution:—

**Resident Medical Officers.**

Poplar Hospital for Accidents, Senior House Surgeon—£100, board, &c.
Evelina Hospital for Children, Junior Medical Officer—£50, board, &c.
Birmingham General Hospital, Assistant House Surgeon—Board, &c.
Chester General Infirmary, Visiting Surgeon—£80, board, &c.
Weston-super-Mare Hospital, House Surgeon—£50, board, &c.
Ipswich Hospital, House Surgeon—£80, board, &c.
Morpeth Dispensary, House Surgeon—£120, rooms, &c.
Wolverhampton General Hospital, Assistant—Board, &c.
Bradford Infirmary, Junior House Surgeon—£50, board, &c.
Denbighshire Infirmary, House Surgeon—£85, board, &c.

**Non-Resident Medical Officers.**

Great Northern Central Hospital, Surgeon to the Out-patients.
University College, London, Surgical Registrar.
St. Bartholomew's Hospital, Physician, Accoucher and Lecturer on Midwifery, and Assistant Accoucher.
Royal Westminster Ophthalmic Hospital, Clinical Assistants.
Glasgow University, Examiner in Medical Jurisprudence—£30.

---

Christmas parcels were received last week from Miss Emily Jones, Mildmay, and Miss McEwen, Richmond. Parcels received this week will be acknowledged in our next; also all the bed-jackets sent in for competition.

---

The Friendly Societies of Suffolk have seriously taken up the question of a Convalescent Home, and the following resolution was lately passed with enthusiasm: "That in the opinion of this meeting of representatives of the United Friendly Societies of Bury St. Edmund's, the scheme for the establishment of a Convalescent Home in connection with the Bury societies, and also those of the whole county, if practicable, is considered advisable, and that this committee represent to their respective societies the result of their deliberations, and accept, with thanks, Bro. Jones's offer to explain his scheme to the respective committees of each society." The scheme is this: The lodges expressing a desire to co-operate would be able either to participate in the home by subscribing for tickets, or by forming a distinct fund from the members' subscriptions, which need not exceed 1d. per quarter. It was shown that as a rule, if the working of other homes might be taken as a criterion, only one member in 200 required the advantages of the home once a-year, and the cost of maintenance was generally considered to be 10s. 6d. per patient per week, so that a lodge of 200 members would require to raise a sum of 2d. per member per year to obtain the benefit for one of its members for three weeks. The question of accommodation was not difficult to work out, and it was shown on a calculation that the provision of six beds would be sufficient accommodation for 120 patients in the year, the average length of the stay of each being about two-and-a-half weeks.

---

The Fancy Fair given by Madame Oliver Christmas, on Tuesday, in aid of the Samaritan Fund of the Middlesex Hospital, was a charming function. True, it was "your money or your life" once you got inside the doors, but the demand was made in so many amusing and interesting ways that one's pockets emptied as if by magic. The stall-holders were in fancy costume, and consisted of Miss Harriett Jay, Miss Letty Lind, little Miss Minnie Terry, and other celebrities from stage-land. Naturally there was no bashfulness about these ladies, and their costumes were most picturesque. Laughter and fun, recitations and songs, filled in the time, and the refreshment-room, where smoking was permitted, was as crowded as the sale rooms. The most charitable people in the world are reported to be actors and actresses, and certainly it looked like it on Tuesday and Wednesday.

---

A few years ago there was no home for children with open wounds or abscesses, and long cases, such as hip-disease, could not be kept in the hospitals, and were therefore sent to their own unfavourable dwellings. Now there are several Homes—one just started at Witney, in Oxfordshire, which is under the patronage of Canon Foxley Norris, the Rector of Witney. At present there are three little patients there from Great Ormond Street, the eldest four years and the youngest ten months. There is accommodation for a few children of the better class. Another Home at Caldicote House, Bushey Heath, though only started a year ago, has been most successful, and its bright, pleasant rooms are always full of poor wee invalids. Both homes charge a small sum only for each patient, and Caldicote House is badly in need of £20 just now for a bath-room.

---

In the discussion following Miss Wilson's paper before the Hospitals Association, Mr. Thomas Ryan compared the condition of things in a workhouse infirmary to those in a well managed London hospital. Though he knew the nursing in the former was necessarily not so scientific as in the latter, still he held the great difference was indefensible. The panacea for all hospital ills was supposed to be a central authority, but nothing could be more condemnatory of the centralization of authority than the state of the present workhouse infirmaries. Miss Evans, lady guardian of the Strand Union, gave some of her experiences, and said she hoped the time would come when there would be no more pauper nurses. Referring to the medical staff of the Strand Union Miss Evans said there was one medical officer, non-resident, who attended about two hours daily, a period quite insufficient for visiting between 300 and 400 patients. The matron of the Chelsea Infirmary said that at the present time she had 34 to 36 nurses of at least a year's training for some 400 patients; she held that the wards of Chelsea Infirmary would bear comparison with those of any hospital. She ventured to express disapproval of lady guardians, so far, at all events, as infirmary management went. Lieutenant-Colonel E. Montefiore drew a dire picture of the Scotch infirmaries.

---

Dr. J. S. Bristowe complimented Miss Wilson on the excellent paper she had read, and stated that his knowledge of infirmaries was confined to Marylebone and Paddington. At one of these there were only two medical officers and a few assistants, a very small staff, in his opinion, for what had to be done. Dr. Bristowe agreed in Miss Wilson's view that the nurses should be under the superintendence of a trained matron, and paupers should not act as nurses. Sir William Moore said he did not think it was fully enough remembered what infirmaries were, compared with what they are. This gave hope for the future.

---

The Chairman, Mr. Rathbone, M.P., said he wished to deal with two points; the first, a very important one—the relation of the matron to the nursing staff. He had attended a meeting at the London Hospital, and had heard it stated that Miss Nightingale had fifteen years ago laid down the rule that authority over the nursing staff should be concentrated in the matron. That principle was laid down by Miss Nightingale not fifteen, but thirty years ago, and every year experience proved its wisdom. He did not believe in the good management of any hospital that did not carry that principle out. To expect the matron to be subject to interference from without was as foolish and destructive of discipline as would be the interference with a General in the line of battle. In both cases life was at stake, and unless responsibility were concentrated, in both instances death, and not life, would be the result. With a view to strengthening Miss Wilson's hand in her efforts, Mr. Rathbone moved a resolution to the effect that Miss Wilson's paper contained the minimum requirements which should be enforced by the guardians in each union. This was carried *nem. con.*

